# Aquaporins and Neurodegenerative Diseases

**DOI:** 10.2174/157015910791233150

**Published:** 2010-06

**Authors:** Eleonora Foglio, Luigi Fabrizio Rodella

**Affiliations:** Division of Human Anatomy, Department of Biomedical Sciences and Biotechnologies, University of Brescia, Viale Europa 11, 25123, Brescia, Italy

**Keywords:** Aquaporins, CNS, Neurodegenerative Diseases.

## Abstract

Aquaporins (AQPs) are a family of widely distributed membrane-inserted water channel proteins providing a pathway for osmotically-driven water, glycerol, urea or ions transport through cell membranes and mechanisms to control particular aspects of homeostasis. Beside their physiological expression patterns in Central Nervous System (CNS), it is conceivable that AQPs are also abnormally expressed in some pathological conditions interesting CNS (e.g. neurodegenerative diseases) in which preservation of brain homeostasis is at risk.

The purpose of this review was to take in consideration those neurodegenerative diseases in whose pathogenetic processes it was possible to hypothesize some alterations in CNS AQPs expression or modulation leading to damages of brain water homeostasis.

## 1 ALTERED WATER HOMEOSTASIS IN NEURODEGENERATIVE DISEASES

Several million people world-wide are affected by neurodegenerative disease, a heterogeneous group of diseases affecting specific areas of Central Nervous System (CNS) and leading to a gradual and progressive cognitive or movement impairments, depending on the type of neuronal cells undergoing selective degeneration [11]. The majority of these pathological conditions are age-related: their frequency, in fact, has increased in the last decades, together with an increased life expectancy of the individuals [61]. Neuronal damage and oxidative stress (primary events in the development of these kinds of disorders) lead to increased production of pro-apoptotic and pro-inflammatory cytokines as well as altered homeostasis of water, extracellular ions and aminoacid neurotransmitters. In consequence of the damaged brain homeostasis observed in many of these pathologies, some authors hypothesized a possible role for Aquaporin (AQP) family members in their pathogenetic mechanisms. The purpose of this paper was to review the recent findings about the involvement of these water channel proteins in the developmental processes of these neurodegenerative diseases, focusing on Neuromyelitis Optica (NMO), Alzheimer’s Disease (AD), Parkinson’s Disease (PD), Amyotrophic Lateral Sclerosis (ALS) and Transmissible Spongiform Encephalopathies (TSE).

## 2 NEUROMYELITIS OPTICA

Inflammatory demyelinating diseases of the CNS occur throughout the world and are the foremost reason for non-traumatic neurological disability in young, white adults [76]. Neuromyelitis optica (NMO) was first described in the late 19^th^ century by Eugène Devic and the syndrome came to be eponymously called Devic’s disease [15, 16]. NMO is defined as a severe monophasic syndrome that is characterized by near-simultaneous bilateral optic neuritis (ON), acute transverse myelitis and inflammatory demyelination with a selective involvement of the optic nerve and the spinal cord where affects gray and white matter, causing blindness and paralysis [99]. Although NMO has been classified as a subtype of multiple sclerosis (MS) for many years, it is now clear that it has distinct clinical and pathological features. Neuropathologic studies demonstrate that NMO is characterized by distinctive “vasculocentric” pathology with prominent perivascular immunoglobulin deposition (IgG and IgM) and evidence of activation of complement lytic pathway, invoking B cell participation in the disease process [56, 100]. These observations further support an auto-antibody mediated pathogenesis for NMO.

### 2.1 Discovery of Specific Auto-Antibodies Against AQP4 in NMO Patients

In 1998, the observation that patients with NMO frequently had multiple autoimmune disorders and a variety of non organ-specific auto-antibodies prompted a search for a specific auto-antibody marker of NMO. Lennon and colleagues [54] described an IgG, the presence of which was 73% sensitive and 91% specific for clinically defined NMO. The detection of NMO specific serum auto-antibodies, collectively known as NMO-IgG, helps to allows early diagnostic distinction between NMO and MS [54, 56]. Typically, NMO-IgG is detected at the first attack of myelitis or ON, suggesting that the antibody is more likely an integral part of the pathogenesis of the disease rather than an epiphenomenon of the tissue injury, but longitudinal data are limited and preliminary.

Accumulating evidences reveal that NMO-IgG localizes to blood-brain barrier (BBB) [54]: an indirect immunofluorescence study with dual immunolabeling of mouse brain sections with NMO patient’ sera and antibodies specific for the endothelial marker factor VIII, the reactive-astrocyte marker glial fibrillary acidic protein (GFAP) or the extracellular matrix protein laminin, has suggested that the main target antigen is in astrocytes located adjacent to the BBB [54]. In white and grey matter of the cerebellum, midbrain, and spinal cord, NMO-IgG bound in a linear pattern along pial membranes, extending into the Virchow-Robin spaces, the abluminal surface of cerebral microvessels, and the subpial region with a mesh pattern. NMO-IgG bound to an antigen in the abluminal face of cerebral microvessels, an area represented by astrocytic foot processes. Moreover, its partial colocalization with laminin [54] is consistent with the auto-antigen being a component of the glia limitans at the BBB [66]. An independent pathologic study had meanwhile recognized an immunohistochemical pattern of immunoglobulin and complement deposition detected around small vessels of autopsied spinal cord tissues of patients with NMO-IgG [56] that was reminiscent of the pattern of immunostaining of serum from NMO patients to the abluminal surface of penetrating microvessels in the mouse brain substrate.

Recently, the target antigen of NMO-IgG has been identified as the mercurial-insensitive water channel protein AQP4 which is the dominant water channel within the CNS [53].

In astrocytic end-feet, AQP4 is closely associated with the cytoskeleton complex. To exclude a primary effect on one of these cytoskeletal proteins as targets, lysates of the AQP4 and control transfected cell lines were immunoprecipitated with antibodies specific for α-syntrophin, β-dystroglycan, and dystrophin (Dp71) as well as antibodies specific to AQP4. Only NMO-IgG or anti-AQP4 antibodies, but not antibodies to other cytoskeletal elements were able to immunoprecipitate the green fluorecent protein (GFP)-AQP4 fusion protein. Then, to determine whether the NMO antigen is restricted to the CNS, NMO-IgG-positive patients' sera were tested on sections of normal non-nervous mouse tissues. In contrast to the characteristic intense staining of pial and microvascular elements in the brain, NMO-IgG did not bind to any vascular or visceral autonomic neural elements in these organs. The distribution of NMO immunoreactivity showed the water channel protein, AQP4, as a candidate antigen. Moreover, using sera from NMO-IgG positive patients on brain tissue obtained from AQP4 knock-out mice, Lennon and colleagues [53] showed that neither human serum IgG from NMO patients nor rabbit anti-AQP4 IgG bound detectably to the AQP4 knock-out mouse brain tissues (Fig. (**1**)).

Furthermore, to demonstrate selective binding to membranes of AQP4 transfected cells, stably transfected human embryonic kidney cell line (HEK-293) expressing a transgene encoding full-length AQP4 and plasmid encoded GFP were created. Neither patients’ nor controls' IgG bound to the HEK-293 cells that were not transfected with the AQP4 containing vector; however, IgG in the sera of NMO patients stained the plasma membrane of AQP4-transfected cells, consistent with the known expression pattern of AQP4 in cells. In addiction, Jarius and colleagues [41] have confirmed that a cell-based or a novel fluorescence-based immunoprecipitation assay that employs recombinant human AQP4 coupled to GFP has higher sensitivity and specificity for NMO than does the NMO-IgG assay. The increased sensitivity of AQP4 assays compared with NMO-IgG testing suggests that AQP4 is the main, or possibly the only, target for NMO-IgG [41, 96].

Taken together, these discoveries make NMO the first inflammatory demyelinating disorder of the CNS in humans in which a specific target for an immune reaction has been identified. NMO-IgG is now utilized as a clinical serological test for this disease and promises to provide substantial insight into the pathogenesis of NMO. Recent data, in fact, have confirmed that assays for anti-AQP4 antibodies are 91-100% specific for differentiating NMO from conventional MS [100].

### 2.2 AQP4 Auto-Antibodies and NMO Pathogenesis

Recently, widespread absence of detectable AQP4 in spinal cord tissue of NMO lesions has been demonstrated by several groups [65, 80, 85]. Misu and colleagues [65] conducted an immunohistochemical analysis that revealed loss of AQP4 in 90% of the acute and chronic NMO lesions, which were more pronounced in the active perivascular lesions where immunoglobulins and complements were deposited. In addiction, Lucchinetti [56] and Roemer [80] and their groups have independently performed detailed comparative study between NMO and MS lesions: in particular, in contrast to NMO, they revealed that AQP4 immunoreactivity was variable in MS lesions (AQP4 is diffusely increased in the white matter of active lesions, whereas chronic inactive lesions are devoid of AQP4).

Moreover, two pathologic patterns in NMO, both of which were associated with loss of AQP4 immunoreactivity were described [39]. The first and most prevalent lesion pattern involves the spinal cord and optic nerves. Loss of AQP4 occurs in the context of vasculocentric immune complex deposition, active demyelination and vascular hyperplasia with hyalinization. These lesions involve both grey and white matter in the spinal cord. Also in the second, less frequent, lesion type AQP4 loss was associated with vasculocentric IgG and IgM deposits, complement activation, and tissue rarefaction, but there was no evidence of demyelination [80]. In summary, the intriguing report by Roemer and colleagues [80], describing loss of AQP4 in the absence of demyelination or necrosis suggests that binding of antibody to AQP4 may be the initial pathogenic event in NMO lesions.

In a comparable study, Misu and colleagues [65] showed some NMO lesions in which myelinated fibers were relatively preserved despite the loss of AQP4 staining, suggesting that the loss of AQP4 did not reflect necrosis and cell loss. These findings suggested that AQP4 loss may be the initial event in NMO lesions.

The limited access of circulating IgG to the extracapillary space in the CNS would only permit interaction of NMO-IgG with AQP4 at the glia limitans of BBB: in consequence of these findings, many authors suggest the perivascular space as the primary target site of the pathogenic process. Current hypotheses concerning the pathogenesis of NMO are divergent: interaction between NMO-IgG and AQP4 could, in turn, activate complement produced locally by astrocytes [68] or cross-link AQP4, thereby perturbing water homoeostasis in the CNS [53].

On the basis of the immunopathological observations, a cascade reaction initiated by the presence of a peripheral antibody directed against AQP4 activating the classical complement pathway was proposed [100]. Activated macrophages, eosinophils and neutrophils generate cytokines, proteases and free radicals leading to vascular and parenchymal damage. Increased vascular permeability could cause further parenchymal destructive processes *via *secondary ischemia, leading to the liberation of novel antigens that may further extend the immune response.

Other auto-antibodies, in fact, have been found in NMO patient sera and cerebrospinal fluid (CSF) (anti-myelin oligodendrocyte glycoprotein (MOG) antibodies) [12, 31]: these latter auto-antibodies likely represent a response to neo-antigen liberated from dead cells and thus are not the initial cause of NMO [31, 50].

Although it has been suggested that AQP4 autoantibodies cause NMO, probably by inhibiting AQP4, [59, 64] other authors [87] keep a more skeptical view pondering that this causality still lacks definite proof, as NMO has not been produced in experimental animals through administration of the antibody. In addition to that, a number of questions remain to be answered: why other organs which highly express AQP4 (e.g. kidneys, lung, inner ear and intestine) are not affected in the disease; why the lesions are predominantly located in the spinal cord and optic nerves, although AQP4 is ubiquitously expressed throughout the nervous system; what is the preceding event opening the BBB to allow the peripheral anti-AQP4 antibody enter the brain; why a NMO-like phenotype does not follow AQP4 deletion in mice.

## 3 ALZHEIMER’S DISEASE

Alzheimer’s disease (AD) is nowadays the most widespread form of senile dementia in the Western countries. AD is clinically characterised by a gradual onset with a progressive and irreversible cognitive decline: memory impairment appears in the earliest stage of the disease although patients’ motor and sensory functions are usually not affected until later stages [13]. Only the post-mortem microscopic examination reveals the critical features of AD: the progressive accumulation of senile plaques (mainly composed of amyloid-β (Aβ)), neurofibrillary tangles (NFTs) (in which the main component is hyper-phosphorylated tau) and neuropil threads in the brain [19]. All these neuropathological hallmarks cause nervous cell death, cortical and sub cortical atrophy and so damage nervous system functions. AD is considered a systemic disease progressively affecting not only the entorhinal cortex, limbic structures and neocortex [9], but also the basal nucleus of Meynert (the main source of the cortical cholinergic innervations), thalamus and brain stem.

Nowadays, the aetiology of AD remains unknown. It is probable that many concomitant factors (both genetic and environmental factors) are relevant in the AD pathogenesis.

### 3.1 Chronically Imbalanced Cholinergic Neurotransmission and Water Motion in CNS

Chronically imbalanced cholinergic neurotransmission affects [69] and accompanies AD [98]. However, it remained unclear whether imbalanced cholinergic neurotransmission may by itself lead to chronic brain damages and, if it does, which molecular, cellular, and physiological pathways are involved.

To approach this question, Beeri and colleagues [7] used transgenic mice with neuronal over-expression of synaptic acetylcholinesterase (AChE-S) of human origin. These mice display a complex central and peripheral phenotype including fluctuations of brain acetylcholine (ACh) levels, elevated high affinity choline transport, and accelerated stress-related neuropathology associated with progressive cognitive deterioration [86]. Prolonged AChE overexpression occurs in the mammalian brain under psychological stress [62], and various studies provide evidence that such stress can induce BBB disruption [84, 89] via yet unknown mechanisms. The authors showed with brain image analysis significant changes in the brain’s diffusion and perfusion parameters strengthen the notion of alterations in water mobility and BBB integrity under cholinergic imbalances. Similar to animal models of ischemia or stroke with elevated AQP4, AchE-S transgenic mice displayed lower baseline motion of water in CNS [52, 83] than their age-matched controls. AQP4 is expressed at higher cellular levels in these animals, but in a similar number of cells in the transgenic and wild-type mice. The modified AQP4 expression patterns were especially intriguing: changes observed in the expression of ion and water channels could explain the differences in the mobility of water and ions into and out of brain cells and in BBB integrity. AQP4 levels in microvascular endothelium increase in diseases associated with BBB disruption [94] and its absence protects the mouse brain from damages characteristic of BBB malfunctioning [58].

### 3.2 AQP4 and Neurovascular Unit in AD Brains

The blood-brain interface has been described as a functional unit commonly called the neurovascular unit (NVU) [1, 34]. A primary function of this specialized region of the brain is to regulate the ionic composition of the extracellular milieu by altering local cerebral blood flow and transport across the BBB. The NVU is well suited for this role since astrocyte end-feet are highly enriched with potassium channels and water channels [2]. Astrocytes of NVU are known to maintain extracellular potassium concentrations in the brain by a process termed “potassium siphoning” [48, 77]. Thus, it is reasonable to predict that changes in potassium channel (Kir 4.1 channels) function may alter brain homeostasis leading to brain impairment. Water movement *via *the AQP4 water channel localized to astrocyte end-feet maintains osmotic balance and promotes effective potassium siphoning [71].

Many accompanying pathologies probably contribute to progression of AD, including cerebrovascular pathology [75]. Blood flow changes including hypoperfusion have been observed in AD [18]. In addiction, accumulation of Aβ in the cerebral vasculature is associated with cerebral amyloid angiopathy (CAA) and has been reported to be present in 78%-98% of all autopsied AD cases [45]. 

Moreover, arterioles and capillaries in AD brains are morphologically characterized by atrophy, swelling and increased pynocitic vesicles in endothelial cells, atrophy and irregularities of smooth muscle fibres, thickening and local disruption of basement membranes and occasional swelling of astrocytic end-feet [24, 29, 34]. All these observations lend support to the idea that water and nutrient transport may be impaired in AD.

In a recent paper, Wilcock and colleagues [97] studied the effects of amyloid accumulation at cerebral vessels on the NVU, using a transgenic mouse model that show amyloid deposition, tau pathology and neuronal loss. Transgenic mice with high levels of CAA have significant reductions in AQP4 and Kir4.1 positive staining associated with the blood vessels. A potential explanation for the loss of AQP4 and Kir4.1 channels is that they share a common anchoring protein that is affected by vascular amyloid deposition: the Dp71 dystrophin protein, localized on perivascular astrocytes [40]. Dystrophin is associated with dystroglycan, which in turn, associates with syntrophins: genetic deletion of syntrophin has resulted in mislocalization of AQP4 [72] and decreased perivascular Kir4.1 channel expression [25]. Thus, changes in dystrophin levels and function may, in part, explain the loss of AQP4 from the astrocyte end-feet as well as the reduction in levels of Kir4.1 channels in mice that express high levels of CAA. Moreover, in support to this hypothesis, Wilcock and colleagues found that gene expression of Kir4.1, AQP4 and dystrophin were significantly reduced also in human post-mortem diagnosed AD brain with moderate and severe CAA.

Overall, these data point to a critical role for the NVU in AD [34, 105]: vascular amyloid deposition results in mislocalization of AQP4 expression and changes in expression levels of specific potassium channels, both of which are critical components of physiological systems designed to maintain the brain external milieu.

### 3.3 AQP1 and AD

A recent study showed that the expression of AQP1 but not that of AQP4 is augmented in cortical astrocytes of AD brains [78]. Supporting this view, previous clinical studies showed that brain ion and water homeostasis is profoundly disturbed in AD [33]. 

In investigating the relationship between Aβ deposition and astrocytic AQP1 expression in the motor cortex and hippocampus of AD patients, Misawa and colleagues [63] found that the great majority of AQP1-expressing astrocytes were located in close proximity to Aβ plaques in AD brains (Fig. (**2**)). In another study, Dohke and Turner [17] analyzed the molecular interaction between AQP1 and Aβ in a transient expression system of HEK293 cells *in vitro*, reporting AQP1 and Aβ_1-42 _co-localization on the cell-surface membrane and in the cytoplasm, when they were co-expressed in cultured cells.

A number of previous studies showed that astrocytes are activated by Aβ itself in AD brains [70, 101]: these observations suggest the hypothesis that Aβ deposition might cause abnormal brain water homeostasis by interfering with astrocytic water channel function. To evaluate the hypothesis described above, a recent study using combined Western blotting and immunohistochemistry in a selected number of sporadic and familial AD showed that the levels of AQP1 expression are significantly elevated in the frontal cerebral cortex of AD patients. Interestingly, these changes occur at early stages of the disease with minimal deposition or diffuse plaques of Aβ, where reactive astrocytes express a cell-surface punctuate AQP1 immunoreactivity [78]. This study also revealed the lack of uniformity of AQP1 expression when comparing astrocytes: some astrocytes were not stained with anti-AQP1 antibodies, whereas AQP1 expression was marked in others. This may explain the lack of relationship between the number of astrocytes (revealed with anti-GFAP antibodies), and the expression of AQP1 in individual cases. The present findings showing increased AQP1 expression in selected astrocytes may represent a link between well-known arteriolar and capillary abnormalities [23, 46] and specific responses to water transport in perivascular processes of astrocytes in AD.

The pathophysiological role of AQP1-expressing astrocytes in AD brains remains unknown. Increasing evidence indicate that AQP1 acts as a gas transporter (such as O_2_, CO_2_, and NO) [8, 20, 32]. In particular, AQP1, by transporting NO out of endothelial cells and into vascular smooth muscle cells, plays a pivotal role in endothelium dependent vasorelaxation [32]. Because the production of NO and reactive species is markedly enhanced in AD brains [4], the possibility exists that AQP1-expressing astrocytes with a great migratory capacity [93] act as a scavenger of these species in the cortex of AD. However, treatment with hydrogen peroxide did not affect the levels of AQP1 protein expression in human astrocytes culture, suggesting that these stimuli do not directly up-regulate AQP1 expression in astrocytes.

The present findings further support the idea that other abnormalities, including abnormal regulation of mechanisms involved in the control of water (and ion) fluxes, occur at early stages in AD. The recognition of events that precede hallmark lesions of AD is crucial for a better understanding of mechanisms that may favour the development and progression of the disease. Further studies, including the immunohistochemical analysis of various disease stages, high-resolution morphological and cell physiological analysis, are required to evaluate this hypothesis.

## 4 PARKINSON’S DISEASE

Parkinson’s disease (PD), the most common neurodegenerative movement disorder, is characterized by an extensive and progressive loss of dopaminergic neurons in the pars compacta of substantia nigra. One of the pathological hallmarks of PD is the presence of Lewy bodies, intracellular inclusions of aggregated α-synuclein. Although the cause and pathogenesis of selective loss of dopamine neurons and the accumulation of α-synuclein in PD remain elusive, growing lines of evidence from environmental risk factors and early-onset genetics point to a convergence between energy metabolism and the disposal of damaged proteins proteasome system function can significantly contribute to the pathogenesis of PD [22].

### 4.1 Dopamine Regulation of AQP4 Expression

In the case of neurodegenerative diseases, neural stem cells that are present throughout adulthood will proliferate and differentiate into new neurons and ⁄or glia [47, 60]. There is evidence that these adult neural stem cells exhibit properties associated with glia both *in vivo* [26] and *in vitro* [35, 51]: for instance, they express GFAP, a marker for differentiated astrocytes [21]. There is also evidence that changes in the number of GFAP positive cells are involved in neurodegenerative diseases such as PD [60]. The neurotransmitter dopamine (DA) stimulates proliferation of progenitor cells, not only in the striatum, but also in the subventricular zone of the adult brain [92]. In a recent study Kuppers and other authors provide evidence that DA regulates the proliferation of striatal astrocytes in culture and that these dopaminergic effects on proliferation are mediated by AQP4 [49]. The results presented by these authors show a down-regulation of AQP4 expression in striatal glial cells *in vitro* mediated by DA.

However, findings about the role of AQP4 in proliferation are few and contradictory. Whereas Saadoun and colleagues [82] reported no change in the proliferation of astrocytes cultured from transgenic mice lacking AQP4, Nicchia and colleagues [74] found a nearly 70% reduction in the cell number of cultivated astrocytes after short interference RNA (siRNA) treatment with RNA duplexes specific for AQP4. Therefore, this hypothesis needs to be corroborated by *in vivo* lesion studies. In addition, the expression of AQP4 in the lesioned striatum needs to be investigated, considering that in the substantia nigra an increase in AQP4 mRNA following 6-hydroxidopamine (6-OH-DA) lesion has been observed [94].

The observation of a down-regulation of astrocytes proliferation by DA confirms and extends these assumption: neurodegenerative diseases correlated with perturbations of the dopaminergic transmission (such as PD) are linked to changes in the proliferation of astrocytes. These findings imply that modulation of AQP4 could be used therapeutically in the treatment of PD.

### 4.2 Mitochondrial AQP9 in PD Brains

In the field of neurodegenerative diseases there is an intriguing although speculative link between AQP9 and PD [67]. In the brain, this water and solute channel is expressed in astrocytes, brain stem catecholaminergic neurons [6], and in subsets of midbrain dopaminergic and hypothalamic neurons [5]. The observed enrichment of AQP9 in mitochondrial inner membranes could suggest a role in metabolic support of the neurons. In particular, it has been hypothesized that altered mitochondrial AQP9 in dopaminergic neurons may relate to their vulnerability in PD [3]. 

Because of the potential importance of mitochondrial AQP9 expression, Yang and colleagues [104] have systematically examined the predicted functional consequences of such expression. They have focused on functional transport measurements of mitochondrial inner membrane preparations: AQP9 function was studied by measurements of water and glycerol permeabilities in brain mitochondria [10, 90]. Permeabilities from rat brain mitochondria were compared with those from organs not expressing AQP9. Neither water nor glycerol permeability differed in mitochondria from the various tissues: in summary, these results provide functional evidence against a role for AQPs in mitochondria. Nevertheless, if AQP9 expression and activity may represent therapeutic targets to improve the treatment of PD, is to date an unresolved question.

## 5 AMYOTROPHIC LATERAL SCLEROSIS

Amyotrophic lateral sclerosis (ALS) is a neurodegenerative disease characterised by progressive muscular paralysis reflecting degeneration of motor neurones with intraneuronal ubiquitin-immunoreactive lesions in the primary motor cortex, corticospinal tracts, brain stem and spinal cord. Approximately two thirds of patients with typical ALS have a spinal form of the disease (limb onset) and present with symptoms related to focal muscle weakness and wasting. Paralysis is progressive and leads to death due to respiratory failure within 2-5 years. The majority of ALS cases are sporadic, but approximately 10% are hereditary (familial ALS; FALS). Some 15-20% of FALS cases have been associated with dominant mutations in the Cu/Zn superoxide dismutase (SOD1) gene [81].

### 5.1 Reduced Expression of AQP4 in Human Muscles with ALS

To date, the functional role of AQP4 in skeletal muscle tissue has not been fully clarified. In experimentally regenerating myofibers, AQP4 was not expressed under denervated conditions, whereas it was expressed when the muscle was innervated [42, 43]: these findings suggested that the expression of skeletal muscle AQP4 needs a nerve supply at the regenerating stage. However, in human muscles with neurogenic atrophy (e.g. ALS), there has been a very few data on the expression of AQP4; in human muscles with neurogenic atrophy, the expression of AQP4 was down-regulated. In rat muscles with experimental denervation, Jimi and colleagues [43] observed a remarkable and rapid decrease in AQP4 at the mRNA and protein levels confirmed by immunohistochemical findings that reveal a decreased AQP4 staining on the surface of myofibers. This study of Jimi and colleagues showed that changes in the levels of AQP4 mRNA in muscles with ALS and other neurogenic atrophies were essentially the same, although there was a greater decrease of AQP4 in ALS than in other neurogenic atrophies. However, the role of innervation in transcriptional regulation of AQP4 needs to be clarified.

Moreover, it is possible to postulate that AQP4 plays an important role in skeletal muscle cell homeostasis. However, water permeability analysis unexpectedly failed to show any differences between wild type and AQP4 knock-out mice in neuromuscular function, suggesting that AQP4 does not play a significant role in skeletal muscle physiology [57; 103]. A compensation of water channel function by other water channel proteins such as AQP3 [95] is another possibility. Further studies are needed to clarify the mechanism of transcriptional regulation by AQP proteins in denervated muscles.

### 5.2 AQP4 as a Potential Marker of BBB Disruption in ALS Models

For years, investigators working in the field of ALS focused their research around motor neuronal death and subsequent pathways, nevertheless, studies of mutant superoxide dismutase 1 (SOD1) animal models, had revealed that also non-neuronal cells are important players in the disease process [91]. Transgenic mice bearing FALS-associated human SOD1 (hSOD1) mutations show lower motor neuron degeneration and clinical symptoms reminiscent of human ALS [30, 102]. Over-expression of AQP4 at the glio-vascular swollen end-feet it has been observed in the transgenic rats SOD1 [73]: these findings are in agreement with the observation that AQP4 induction in perivascular astrocytes has been correlated to BBB breakdown and could serve as a marker of BBB integrity [88]. This concept was already approached by Kaiser and colleagues [44], who observed a progressive loss of Kir4.1 and a slight increase in AQP4 protein in the mouse ALS model. Kir4 co-localizes with AQP4 channels and clusters around the microvasculature of the spinal cord; together these two proteins buffer K^+^ and water away from the extracellular space to extrude it into blood vessels in the spinal cord [14] (Fig. (**3**)). However, AQP4 over-expression is also noted in stacked glial processes directly in contact with motor neurons [73]. This observation, associated with findings that impaired K^+^ buffering through loss of Kir.4 channel [44], could give an explanation to the understanding of the paracrine toxicity of motor neurons that is typical of ALS.

Taken together, these data corroborate evidences of AQPs involvement in BBB disruption in animal models of ALS. This disruption may contribute to a vicious cycle of the disease process by spreading inflammation within the CNS and so weakening susceptible neuronal functions. Further confirmations have to be obtained from human disease samples and may lead to therapeutic strategies fog restoring the BBB integrity in ALS.

## 6 TRASMISSIBLE SPONGIFORM ENCEPHALOPATHIES

Prion diseases, also called TSEs are cerebral amyloidosis characterized by deposition and accumulation of a pathologic protein (PrPres), neuronal loss, gliosis, and spongiform change [27, 36]. The spongiform change, a cardinal lesion in these diseases, consists of the formation of rounded vacuoles within the neuropil that are swollen neuronal and astrocytic cell processes with disrupted membranes, often in contact with synaptic structures [55]. Little is known about the mechanisms which participate in the vacuolization of the neuropil [28]. However, spongiform change is likely the result of abnormal membrane homeostasis and increased water content within swollen cell processes. Therefore, exploration of mechanisms regulating water homeostasis seems to be a promising step toward increased understanding of the pathogenesis of TSEs.

### 6.1 AQP1 and AQP4 Brain Expression in TSE

Enhanced expression of AQP4 in Creutzfeldt-Jakob disease (CJD) was first reported by Iwasaki’s laboratory [37]. Furthermore, Rodriguez and colleagues [79] analyzed AQP1 and AQP4 expression in the cerebral cortex of CJD patients compared to that in cases with AD and diffuse Lewy body disease (DLB). Significant increased expression levels of AQP1 and AQP4 were seen in CJD, but not in advanced AD and DLB cases. Immunohistochemistry revealed that AQP1 and AQP4 were expressed in astrocytes in diseased cases. Moreover, the findings of the present study indicate that increased AQP4 immunoreactivity is not restricted to the early stage of CJD but also occurs at later stages in the lesions of status spongiosus.

In a similar study, instead, Iwasaki and colleagues [38] compared cases of subacute spongiform encephalopathy (SSE) and panencephalopathic (PE)-type CJD. Increased AQP4 immunoreactivity was observed in all these patients, particularly in the cerebral neocortex and cerebellar cortex. AQP4 immunoreactivity was present in the cell bodies and processes of protoplasmic astrocytes in SSE and around cell bodies and processes of hypertrophic astrocytes in PE-type CJD.

Together, these findings show increased expression of water channels in the brain in human and animal prion diseases that may have implications in the regulation of water transport in astrocytes and may account for an imbalance in water and ion homeostasis. Although change in AQPs expression may be a secondary change of TSE pathologies, it may reflect important aspects of astrocytic pathology associated with these diseases.

## CONCLUSIONS

Water transport is a fundamental process contributing to human physiology and pathophysiology. The importance of AQPs in the physiology of water and solute movement in the CNS is now clear. Therefore, targeted pharmacological modulation of water and solute transport using AQPs would appear to provide novel opportunities for therapeutic interventions in a variety of human disorders involving CNS, like neurodegenerative diseases.

## Figures and Tables

**Fig. (1):**
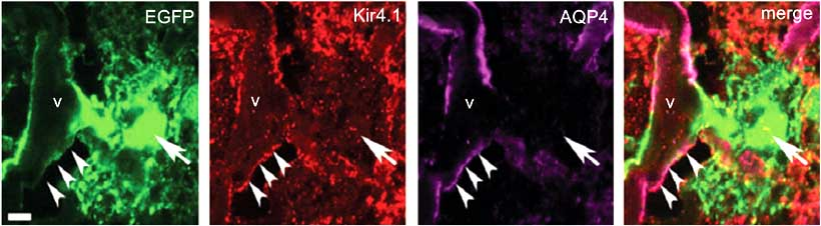
**Immunofluorescence reveals NMO-IgG co-localization with AQP4 in mouse tissues. (A)** Brain Virchow-Robin space (pial-astrocyte interface): NMO-antigen (green, fluoresceinconjugated IgG), AQP4 water channel protein (red, rhodamineconjugated IgG); merged images yellow. **(B)** WT mouse brain binds NMO-IgG (panel 1) in a pattern that is indistinguishable from its binding of AQP4-specific IgG (panel 4); pia, subpia, and microvessels are stained. However, AQP4 knock-out mouse brain (null) did not bind NMO-IgG (panel 2), and the serum of a control patient who had neuropsychiatric disease did not bind to WT brain (panel 3). Figures from Lennon *et al*., [53], reprinted with permission of The Rockefeller University Press.

**Fig. (2):**
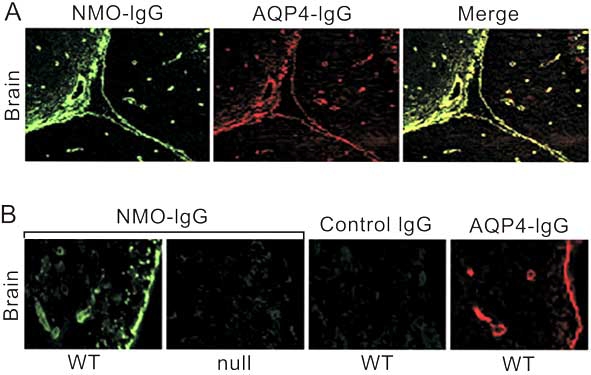
**Multipolar fibrillary astrocytes express AQP1 in the cerebral motor cortex of AD. (a)** AQP1 (*brown*) and Aß (*red*). Multipolar fibrillary astrocytes expressing an intense AQP1 immunoreactivity are often located on the top of Aß deposition; **(b)** Aß (*red*) in the hippocampus of the patient with AD. Figures from Misawa *et al*., [63], reprinted with permission of Springer.

**Fig. (3):**
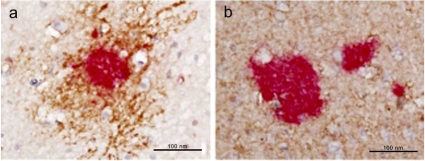
**Co-localization between Kir4 and AQP4 in adult mouse spinal cord.** Kir4.1 immunolabeling (panel 2, red) showed clustering on astrocytic endfeet (panel 1, green). (panel 3) AQP4 (magenta) immunoreactivity on EGFP/GFAP-positive astrocytes predominantly labeling astrocytic endfeet. (panel 4) Merged images of (panels 1-3). Arrowheads point to an astrocytic endfoot co-labelled by Kir4.1 and AQP4. Note that the astrocytic cell body (arrow) was only weakly labelled by Kir4.1 and AQP4, whereas the expression of both membrane proteins was predominantly targeted to the astrocytic network and astrocytic endfeet. Figures from Kaiser *et al*., [44], reprinted with permission of Wiley Interscience.
